# Implementation of the Community Model at the Adult Psychiatric Clinic of Mytilene, Lesvos, Greece

**DOI:** 10.1192/j.eurpsy.2026.10774

**Published:** 2026-07-31

**Authors:** N. Kourti

**Affiliations:** Adult Psychiatric Clinic of Mytiline, Mental Health Unit (General Hospital of Mytiline), 2nd Regional Health Authority of Piraeus and Aegean Islands, Mytiline, Greece

## Abstract

**Introduction:**

The initiative to implement the community model in the Adult Psychiatric Clinic of Mytilene was driven by the goal of establishing Social Psychiatry on the island of Lesvos. The objective was to develop the necessary institutions and structures to operate the Psychiatric Sector as a model of Community Psychiatric Care, promoting holistic, patient-centered mental health services.

**Objectives:**

Significant resistance was encountered, particularly from nursing and paramedical staff, who were hesitant to join therapeutic teams and shift from traditional hierarchical approaches to cooperative work with patients. Hospital staff, also expressed discomfort and fear about the presence of psychiatric patients moving freely within the hospital grounds, even with professional accompaniment.

**Methods:**

A multi-layered approach was adopted, focusing on both systemic reform and individual therapeutic engagement: 1) assignment of a Referral Person to each patient, 2) Therapeutic and Support Groups (weekly special staff group, monthly general staff group, weekly inpatient community group, weekly outpatient group, biweekly supervision group. 3) integration of patients into individual, group, and family therapy programs, 4) establishment of a Day Center to support daily structure and rehabilitation, 5) creation of a Mobile Mental Health Unit for community outreach, 6) implementation of mental health promotion activities in the broader community.

**Results:**

The implementation led to transformative changes in care delivery and outcomes. As a result, we observed significant improvement in the following areas: 1) active patient participation in the therapeutic process, 2) enhanced collaboration between staff and patients, 3) creative employment as a therapeutic tool, 4) promotion of personal responsibility in patients, 5) encouragement of initiative from both staff and patients, 6) development of a sense of community within the clinic, 7) significant reduction in involuntary hospitalizations, 8) use of minimal necessary medication, 9) decreased use of restraint methods, 10) improved patient functionality and faster, more complete social rehabilitation, 11) notable reduction in stigma, both within the institution and the community.

**Conclusions:**

Over the past three years, the community psychiatric model implemented at the Adult Psychiatric Clinic of Mytilene has demonstrated that even within a biologically-oriented clinical setting, the need for coercive practices can be significantly reduced. This model fosters a holistic, participatory therapeutic environment, where patients are treated as equal members of the therapeutic relationship and are supported in reclaiming their roles and responsibilities within their families and communities. It represents a practical and effective step toward realizing the principles of Social Psychiatry and community-based care.

**Disclosure of Interest:**

None Declared

